# High Rate Deposition of Piezoelectric AlScN Films by Reactive Magnetron Sputtering from AlSc Alloy Targets on Large Area

**DOI:** 10.3390/mi13101561

**Published:** 2022-09-21

**Authors:** Stephan Barth, Tom Schreiber, Steffen Cornelius, Olaf Zywitzki, Thomas Modes, Hagen Bartzsch

**Affiliations:** Fraunhofer Institute for Organic Electronics, Electron Beam and Plasma Technology FEP, 01277 Dresden, Germany

**Keywords:** AlScN, aluminum scandium nitride, piezoelectric thin films, piezoelectric, ferroelectric

## Abstract

This paper reports on the deposition and characterization of piezoelectric Al_X_Sc_1-X_N (further: AlScN) films on Si substrates using AlSc alloy targets with 30 at.% Sc. Films were deposited on a Ø200 mm area with deposition rates of 200 nm/min using a reactive magnetron sputtering process with a unipolar–bipolar hybrid pulse mode of FEP. The homogeneity of film composition, structural properties and piezoelectric properties were investigated depending on process parameters, especially the pulse mode of powering in unipolar–bipolar hybrid pulse mode operation. Characterization methods include energy-dispersive spectrometry of X-ray (EDS), X-ray diffraction (XRD), piezoresponse force microscopy (PFM) and double-beam laser interferometry (DBLI). The film composition was Al_0.695_Sc_0.295_N. The films showed good homogeneity of film structure with full width at half maximum (FWHM) of AlScN(002) rocking curves at 2.2 ± 0.1° over the whole coating area when deposited with higher share of unipolar pulse mode during film growth. For a higher share of bipolar pulse mode, the films showed a much larger c-lattice parameter in the center of the coating area, indicating high in-plane compressive stress in the films. Rocking curve FWHM also showed similar values of 1.5° at the center to 3° at outer edge. The piezoelectric characterization method revealed homogenous d_33,f_ of 11–12 pm/V for films deposited at a high share of unipolar pulse mode and distribution of 7–10 pm/V for a lower share of unipolar pulse mode. The films exhibited ferroelectric switching behavior with coercive fields of around 3–3.5 MV/cm and polarization of 80–120 µC/cm².

## 1. Introduction

In 2009, Akiyama et al. first reported on the Sc doping of AlN films by the cosputtering process [1]. These Al_X_Sc_1-X_N films exhibited significantly increased piezoelectric properties depending on the Sc concentration with a maximum at 43 at.% Sc (Al + Sc = 100 at.%). Since then, research and development on AlScN films has increasingly gained attention from research groups as well as industry worldwide. The focus is mostly on MEMS [2,3] and acoustic wave applications [4,5], but energy harvesting applications [6,7,8] are also gaining traction.

Furthermore, Fichtner et al. demonstrated in 2019 the possibility of ferroelectric switching in AlScN films with scandium concentrations between 27 at.% and 43 at.% [9]. The ferroelectric switching occurs at coercive fields of 2–5 MV/cm, with higher Sc concentrations showing reduced necessary fields for switching but in turn also lower polarization values from above 100 µC/cm² to around 80 µC/cm². Further research by different groups worldwide is ongoing [10,11].

One aspect that still proves very challenging for the development of AlScN deposition processes is the increasing probability of the formation of abnormally oriented grains (AOG) at the grain boundaries, especially at higher Sc concentrations [12], tensile film stress [5] and higher film thickness [12,13]. This limits the process window in which films with good piezoelectric properties can be fabricated as well as the possible applications. Therefore, to further extend the material and process range and enable new applications, additional process parameters and adjustment options are necessary. One such process parameter is the pulse mode of powering in reactive pulse magnetron sputter processes. Usually, the configuration can either be unipolar or bipolar pulse mode. This results in different plasma conditions and consecutively film properties. By developing and using a unipolar–bipolar hybrid pulse mode, Barth et al. could freely influence film properties of cosputtered AlScN films from Al and Sc targets in a much wider range, realizing highly oriented films with thicknesses of up to several 10 µm [14]. This paper investigates if such pulse mode variation can be used to deposit highly oriented Al_0.7_Sc_0.3_N films from Al_0.7_Sc_0.3_ alloy targets with a high deposition rate and good homogeneity on a large area with adjustable film properties in a wider range. This would offer new application opportunities, e.g., in thicker films of several µm for energy harvesting or ultrasonic devices or as stress-optimized thin films in multilayer stacks.

## 2. Materials and Methods

### 2.1. Film Deposition

**Figure 1:**
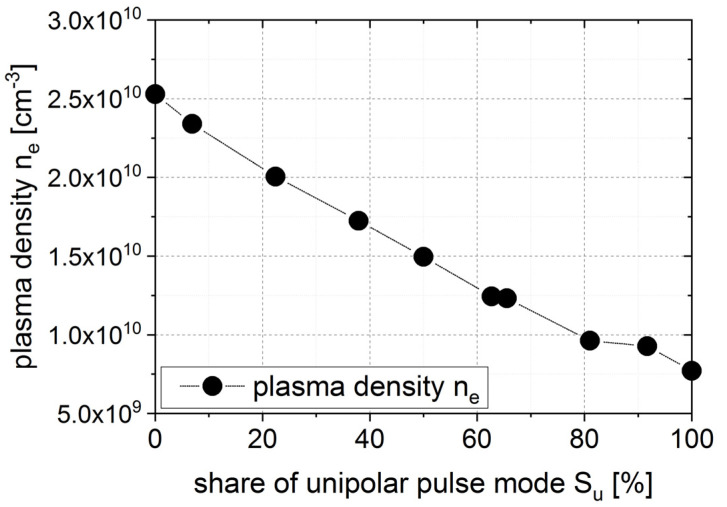
Plasma density in dependence of share of unipolar pulse mode in unipolar–bipolar hybrid pulse mode operation (example of Si process, adapted from [14]).

The films were deposited using a reactive pulse magnetron sputter process described in [14]. The double-ring magnetron DRM 400 uses two concentric targets, whose discharges overlap to deposit homogenously on a wide area. Thus, films can be deposited with a very high film growth rate of 200 nm/min with film thickness homogeneity of up to 0.5% on a Ø200 mm area. Film thicknesses can be several tens of microns. By using the pulse unit UBS-C2 developed by FEP and standard DC power supplies, the pulse mode of the pulse magnetron sputtering can be adjusted as unipolar, bipolar or as a unipolar–bipolar hybrid pulse mode. In unipolar pulse mode, a pulsed dc voltage is applied between each of the two targets acting as cathodes and the separate hidden anode. In bipolar pulse mode, the two targets are alternately the cathode and the anode. In hybrid pulse mode, different shares of unipolar (S_u_) or bipolar (S_b_) pulse modes can be applied in a period of 1 ms to influence plasma parameters. The share of unipolar pulse mode is defined as the ratio between the time fraction of unipolar mode t_u_ with respect to the total time of one cycle of unipolar–bipolar hybrid pulse mode, i.e., Su = t_u_/(t_u_ + t_b_). The plasma parameters can be adjusted in a wide range between both pure pulse modes, as shown in the plasma density measurements of an example process of Si sputtering in Figure 1.

In the case of AlScN film depositions, AlSc targets (3N5) with Sc contents of 30 at.% were used. Argon and nitrogen (5N) were used as inert and reactive gas, respectively. The gas flow was between 20 and 60 sccm, and the pressure was between 0.3 Pa and 0.8 Pa. A closed-loop reactive gas control was applied to stabilize the process in the transition mode [15]. The share of unipolar pulse mode in hybrid pulse mode operation was investigated between 60% and 90% of the period. The chamber base pressure was 2 × 10^−7^ mbar. Before coating each sample, a precleaning of the substrate surface was performed using rf bias etching in an Ar atmosphere. There was no additional substrate heating or cooling beyond water cooling of the substrate platform applied.

### 2.2. Characterization

Film composition was determined by energy-dispersive spectrometry (EDS) of X-ray (Octane Elect Plus, EDAX, Pleasanton, CA, USA) using 10 kV accelerating voltage and the aluminum and scandium Kα lines for quantification.

Film stress measurements were performed using the wafer curvature method based on Stoney’s equation. The measurements were carried out with a surface profiler, P15-Ls (KLA Tencor, Milpitas, CA, USA).

The XRD characterizations were carried out in a Bruker D8 Discover diffractometer equipped with a Göbel mirror for Cu-Ka parallel beam geometry and a 1D LynxEye^TM^ XET semiconductor detector (Bruker AXS, Karlsruhe, Germany). θ–2θ scans in 1D detector mode were used to characterize lattice parameters of the wurtzite structure. Film quality in terms of crystallographic orientation was investigated by evaluating the (002) rocking curve FWHM in 0D detector mode. For calculation of the wurtzite lattice parameter c, the (002) peaks in θ–2θ scans were used. Peak fitting including a Cu-K_α1/α2_ doublet correction followed by a correction for instrumental 2θ alignment errors was carried out to extract the exact 2θ peak positions from the raw data.

The piezoresponse force microscopy (PFM) measurements were carried out using an AFM NX 20 from Park Systems (Suwon, Republic of Korea). Silicon wafers coated with a Ti/Pt seed layer and bottom electrode for grounding connection were used as the substrate for AlScN layers. An ac voltage with an amplitude of 10 V and 17 kHz frequency was applied to a platinum-coated AFM tip (Spark 350 Pt) to measure PFM amplitude and phase of inverse piezoelectric effect in off-resonance mode.

The piezoelectric and ferroelectric characterization was performed using a double-beam laser interferometer (DBLI) of aixACCT systems GmbH (Aachen, Germany). This method uses laser interferometry to measure surface displacement of the sample depending on applied electrical voltage. By using a double-beam configuration, substrate bending can be considered. The effective piezoelectric coefficient d_33,f_ of the film material can be calculated by measuring at different electrode pad sizes and correcting using a geometric factor f(r), which is a function of the ratio of the pad size to substrate thickness and substrate Poisson’s ratio. Besides d_33,f_, this also allows calculation of transverse effective piezoelectric coefficient e_31,f_. The method is described in further detail in [16].

## 3. Results and Discussion

### 3.1. Composition and Structure of AlScN Films

**Figure 2:**
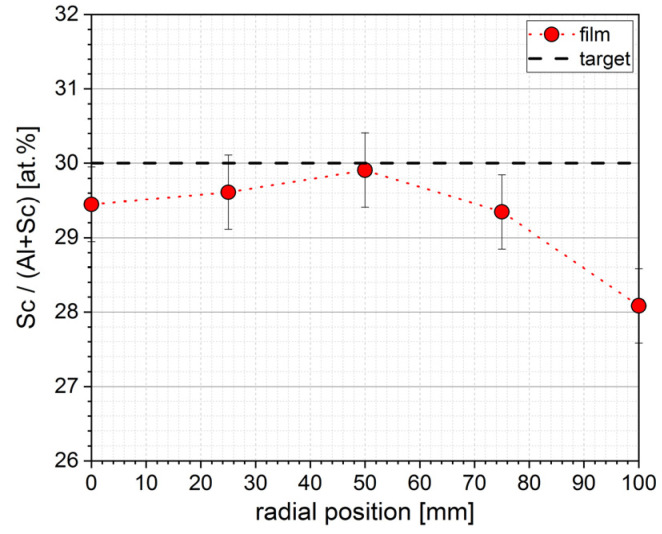
EDS measurement of Sc content in AlScN and comparison to the Al_0.7_Sc_0.3_ target composition.

The first coatings were performed directly on Si substrates, and the film thickness distribution was ±1.5% at Ø180 mm and ±3% at Ø200 mm. The film composition was determined by EDS. The results are shown in Figure 2. The Sc content of the Al_X_Sc_1-X_N films was slightly below that of the nominal target composition as given by the manufacturer, especially at a longer distance from the symmetry axis of the magnetron, going as low as 28 at.% Sc at the radial position 100 mm, while being around 29.5 at.% Sc in the inner coating area. These (minor) variations can be attributed to geometric effects such as shadowing of particle flux by the outer plasma shields and the difference in angular distribution of sputtered Al and Sc atoms. The latter effect is expected to be more pronounced at low target–substrate distances (TSD 90 mm) and low pressure, i.e., a high mean free path of atoms.

**Figure 3:**
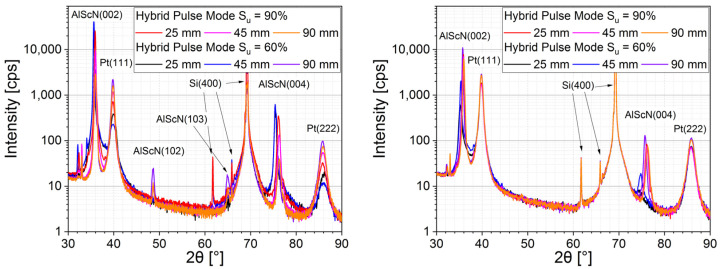
θ–2θ scan of 2.5µm (**left**) and 0.9 µm (**right**) AlScN films at different radial positions (25 mm, 45 mm, 90 mm); peaks labeled as Si(400) are the Si substrate peaks by Cu K_α1/2_ (69.2°), Cu K_β1_ (61.7°) and Cu W L_α1_ (65.9°); the peaks at 32.2° and 34.2° are the AlScN(002) Peaks by Cu K_β1_ and Cu WL_α1_, as well as Si(200) by Cu K_α1_ at 33.0°, respectively.

Figure 3 shows the θ–2θ scans of AlScN with 2.5 µm and 0.9 µm thicknesses on Si/Ti/Pt with a 5 nm Ti and 40 nm Pt seed layer for different pulse modes and radial positions. All films were highly c-axis-oriented, with (002) peaks being the main intensity by far. The 2.5 µm-thick films deposited with a more unipolar pulse mode (S_u_ = 90%), revealing that there were also weak peaks of (102) and (103) orientation present, although those had a very small intensity of 1:3500 relative to the (002) peak. On the other hand, the films deposited with a lower share of unipolar pulse mode (S_u_ = 60%) exhibited no (102) or (103) peaks in the inner radial positions, while they appeared only at the edge of the coating area (r = 90 mm), with intensity ratios to the (002) peak being 1/1000 and 1/2000, respectively. The thinner films with 900 nm thickness, on the other hand, exhibited no visible (102) or (103) peaks, regardless of pulse mode or radial position, indicating a high crystalline quality over the whole coating area for the thinner films, similar to results for thickness-dependent occurrences of AOG reported in [12].

**Figure 4:**
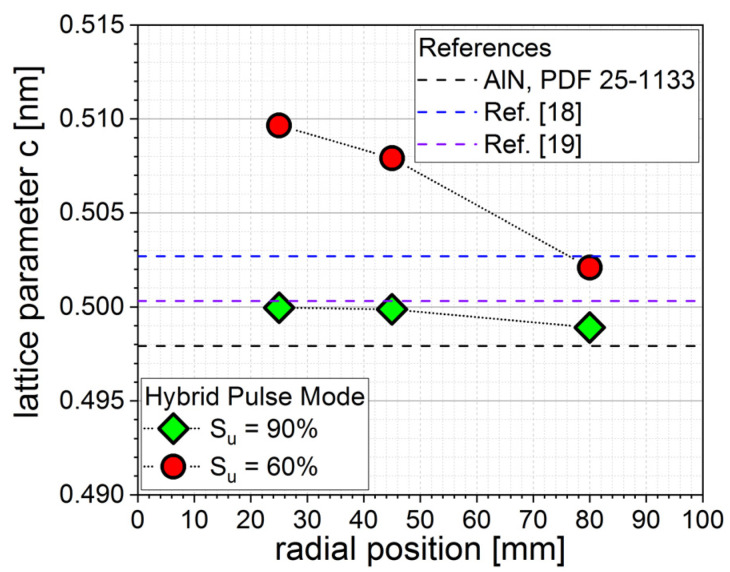
Calculated c-lattice parameter of 0.9 µm films deposited with different hybrid pulse modes depending on radial position (PDF 25-1133; theoretical AlN lattice parameter c = 0.49792 nm, as well as calculated values for Al_0.7_Sc_0.3_N from [18,19] for comparison).

From the 2θ position of AlScN (002) peaks in Figure 3, the c-lattice constant was calculated as described in the Methods section. Film stress, e.g., due to energetic particle bombardment during layer deposition, results in a shift of the c-lattice parameter compared to stress free values [17]. For c-axis-oriented films, the lattice parameter c increased with increasing in-plane compressive film stress. Additionally, besides the effect due to film stress, the lattice parameters a and c of AlScN increased with the incorporation of scandium into the wurtzite structure. The lattice parameters of AlN are a = 0.31114 nm and c = 0.49792 nm according to ICDD PDF-25-1133. According to calculations by other groups, the a-axis lattice parameter of Al_0.7_Sc_0.3_cN should be increased compared to that of AlN by ca. 4.4% to 0.3248–0.3250 nm, and the c-axis lattice parameter should increase by ca. 0.5–1% to 0.5003–0.5027 nm [18,19]. The calculated c-axis values from XRD measurements for different pulse modes and radial positions are shown in Figure 4. As can be seen, the mostly unipolar pulse mode (S_u_ = 90%) was almost homogenous along the whole radius. Film stress, as calculated with the wafer curvature method on a reference sample, i.e., an AlScN-coated 8 inch Si wafer without any electrode layers, was homogenous over the deposition area and around −700 MPa, therefore compressive. On the other hand, the pulse mode with a lower share of unipolar (S_u_ = 60%) was more inhomogeneous, with a 2% bigger c axis in the wafer middle compared to the edge and thus a much higher compressive stress of above 1.5 GPa. This is in line with previous results, revealing higher plasma density near the substrate and higher energetic bombardment of the substrate at a higher share of bipolar pulse mode (see Figure 1, [14]).

**Figure 5:**
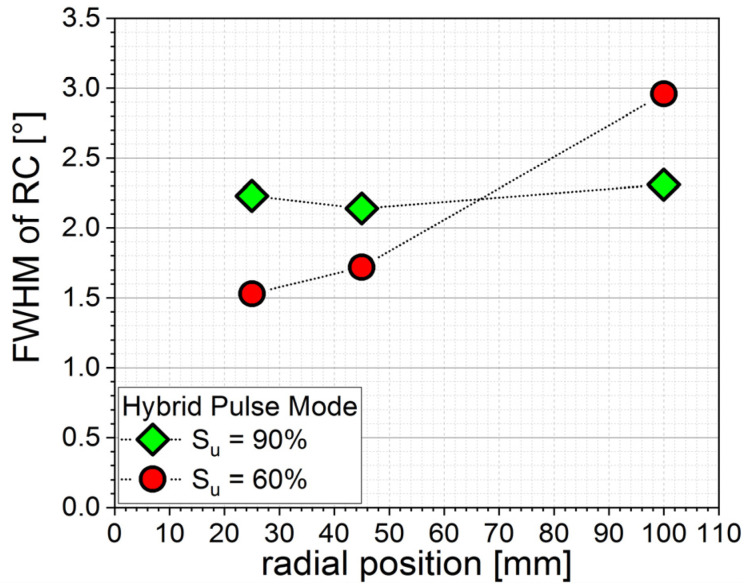
FWHM of (002) rocking curve of 2.5 µm films depending on radial position and pulse mode.

The rocking curves of (002) peaks shown in Figure 5 exhibited a similar behavior of radial dependencies. The films deposited at pulse mode S_u_ = 90% had homogenous values of around 2.1–2.3° over the whole deposition range. In contrast to this, films deposited with a higher share of bipolar pulse mode showed a significant increase in FWHM from 1.5° in the middle, where energetic particle bombardment during film growth was highest, to 3° at the outer position, where energy bombardment was much lower. This is also in line with Figure 3, where the 2.5 µm-thick film deposited at pulse mode S_u_ = 90% showed a homogenous occurrence of, although barely visible, (102) and (103) peaks, whereas the film deposited at pulse mode S_u_ = 60% exhibited them only at the outermost position.

### 3.2. Piezoelectric and Ferroelectric Properties

The piezoelectric properties were characterized on 1.5 mm-thick 4 inch Si wafers to take substrate deformation into account [16]. The bottom electrodes were 5 nm Ti and 40 nm Pt, and the top electrodes were 100 nm Al with different electrode diameters from Ø0.5 mm to Ø2 mm for calculation of d_33,f_ to take pad size effect into account.

**Figure 6:**
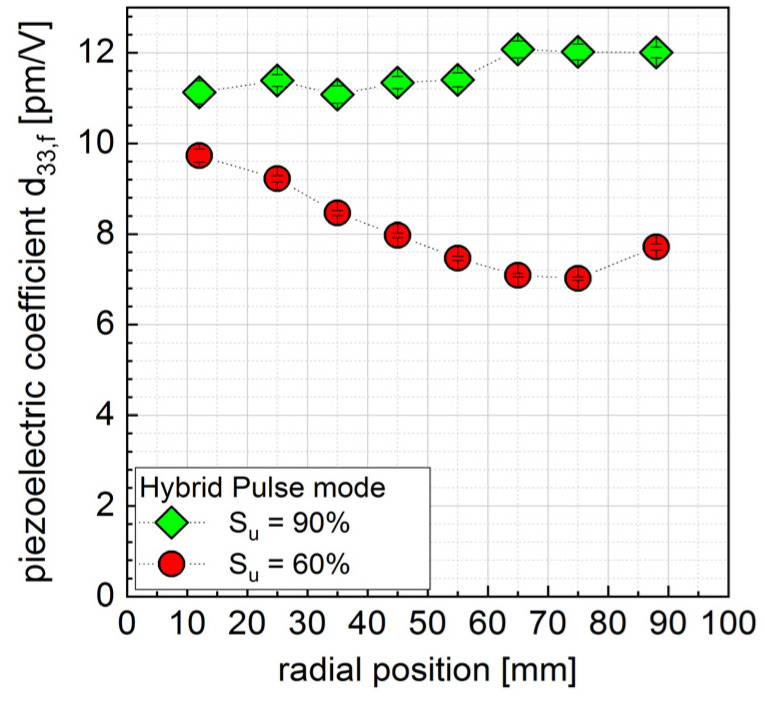
Dependency of d_33,f_ of 1 µm AlScN films on radial position and share of unipolar pulse mode S_u_.

The material parameter d_33,f_ of 0.9µm AlScN films is shown in Figure 6. Every data point refers to a least 20 single electrodes. These electrodes are within < ±5 mm distance to the noted radial position in the figure. As can be seen, the films deposited at a more unipolar pulse mode S_u_ = 90% showed a homogeneous radial distribution of around 11–12 pm/V with only a slight increase towards the outer coating area. This conforms with the XRD data, which show homogenous rocking curve FWHM. On the other hand, the more bipolar pulse mode resulted in films, which showed a maximum of d_33,f_ = 10 pm/V in the middle of the deposition area and a decreasing value until reaching 7 pm/V at radial position around ca. 70 mm from the center with a slight increase towards higher radial positions again. The maximum value of d_33,f_ was lower compared to films grown at higher unipolar pulse mode. This is somewhat congruent with the XRD data, as it shows an increase in FWHM of (002) rocking curve towards the edge, although FWHM started at a lower value compared to films deposited with a more unipolar pulse mode.

**Figure 7:**
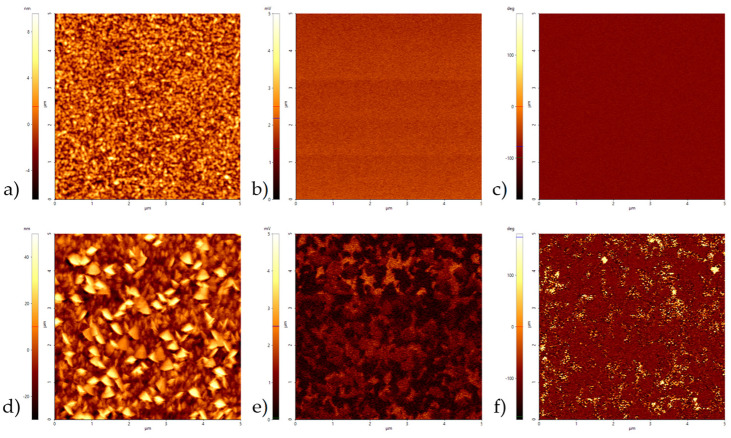
PFM results (area 5 µm × 5 µm) of 2.5 µm AlScN, deposited with S_u_ = 60% pulse mode, at radial positions 25 mm (**a**–**c**) and 100 mm (**d**–**f**): topography (**a**,**d**), piezoelectric amplitude (**b**,**e**) and piezoelectric phase (**c**,**f**).

Figure 7 shows the piezoresponse force microscopy measurements of AlScN deposited with pulse mode S_u_ = 60% at two radial positions. The film surface at the inner radial position of 25 mm was homogenous with an arithmetic average roughness Ra of 1.4 nm, whereas the films at outer position of 100 nm showed a high density of abnormal-oriented grains (AOG) and thus a much higher roughness Ra of 9.7 nm. The measured piezoelectric amplitude showed the same behavior, being homogenous in the inner 25 mm position and having many areas without any piezoelectric activity at the 100 mm edge position of the deposition area. Additionally, the piezoelectric phase of films was unipolar (N-polar) at the inner position and bipolar at the outer position, meaning the film had grown both Al- and N-polar. The cause for this is still under investigation. The most probable cause may be some local distortions during film growth, supported by the higher ratio of AOG on the surface.

**Figure 8:**
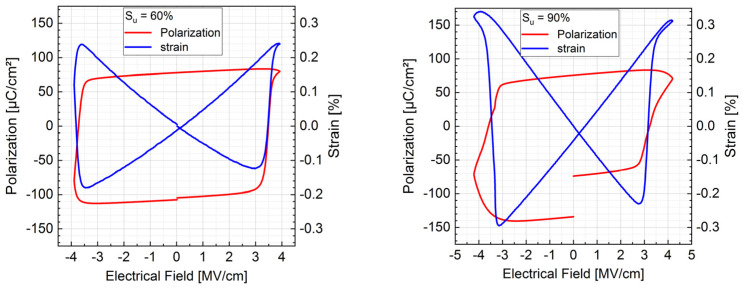
DBLI measurement of polarization and strain of 1µm Al_0.695_Sc_0.295_N deposited with pulse mode S_u_ = 60% (**left**) and S_u_ = 90% (**right**) on Ti/Pt seed layer with Ø0.75 mm Al top electrode (= 0.44 mm²) depending on electrical field, measured at 1 kHz, average of 100 measurements.

The ferroelectric switching of AlScN films deposited with pulse mode S_u_ = 60% and 90% are exemplarily shown in Figure 8 for Ø0.75 mm top electrodes (area of 0.44 mm²). The polarization after switching was ca. 80 µC/cm² on the positive axis for both pulse modes. On the negative axis of applied electrical field, the polarization was 120 µC/cm² for S_u_ = 60% and around 80 µC/cm² with higher leakage current for S_u_ = 90% at negative electrical field. The coercive fields were at ca. 3.1 MV/cm and −3.5 MV/cm for S_u_ = 60% and ca. 3 MV/cm and −3.3 MV/cm for S_u_ = 90%, respectively. This is in line with the values reported by Fichtner et al., where AlScN with 27 at.% or 32 at.% Sc was reported to have a polarization of above 100 µC/cm² with coercive fields of around 4 to 5 MV/cm or 3 to 4 MV/cm, depending on the sign of electrical field, residual stress and Sc concentration [9]. Furthermore, Giribaldi et al. reported an effect of the electrode material on the Pt-AlScN-Al layer stack [20], a layout similar to this paper. They reported the effect of asymmetry of electrical properties as resulting from the combination of a Schottky junction at the Pt–AlScN interface and an ohmic contact at the AlScN–Al interface. As an additional factor, since the film stress of our sample was highly compressive, the resulting P-E loop and strain response on the applied electrical field shifted accordingly. Maximum strain at ±4 MV/cm for S_u_ = 60% and 90% was 0.25% and 0.35% at positive strain (elongation of c axis) and 0.13%/0.18% and 0.23%/0.29% at negative strain (compression of c axis), respectively. This is in line with the higher piezoelectric activity (Figure 6) and better crystalline quality (Figure 3).

## 4. Conclusions

This paper reports the deposition and characterization of ferroelectric AlScN films with a Sc content of 30 at.%. Film composition was largely homogenous at 29.5 ± 0.5 at.% Sc, with a decrease to 28 at.% at the edge due to geometric effects.

XRD θ–2θ scans revealed a highly oriented structure with high c-axis (002) orientation at all parameters, although thick films with a large share of unipolar pulse mode (S_u_ = 90%) exhibited a small occurrence of (102) and (103) orientations with peak intensities of 1/3500th of (002) peak intensity. Calculation of c-lattice parameters revealed a homogenous distribution for more unipolar process conditions, with more bipolar pulse modes (S_u_ = 60%) resulting in films with a higher c axis, i.e., more compressive stress, at the inner coating area. This is in line with known plasma parameter measurements, meaning higher plasma density in the center for bipolar process conditions. Rocking curve measurements of AlScN (002) peaks confirm this, as they revealed homogenous distribution of FWHM values for the 90% share of unipolar pulse mode of around 2.2 ± 0.1° and a radial distribution for the 60% share of unipolar pulse mode, with 1.5° at the center of the deposition area and 3° at the edge.

Piezoelectric characterization by double-beam laser interferometry showed d_33,f_ values of 11–12 pm/V for films deposited by hybrid pulse mode S_u_ = 90% process conditions and 10–7 pm/V for S_u_ = 60% process conditions with a decrease towards outer coating areas. PFM measurements attribute the overall lower piezoelectric behavior of the films deposited by S_u_ = 60% to a stronger occurrence of AOG and a bipolar phase distribution.

The films showed ferroelectric switching behavior with coercive fields of 3 MV/cm to 3.5 MV/cm for 0.9 µm AlScN films. Polarization of the films reached 80–120 µC/cm², depending on switching direction and pulse mode. The maximum strain at the highest field of 4 MV/cm was around 0.35%, also depending on switching direction and pulse mode.

In short, the influence of pulse powering variation by special unipolar–bipolar hybrid pulse mode on resulting film properties of AlScN was investigated. It was shown that good structural and piezoelectric properties can be achieved with very high deposition rates of up to 200 nm/min on large areas using this hybrid pulse mode operation.

## Data Availability

The data presented in this study are available on reasonable request from the corresponding author.
